# Assessment and Assay Comparison for Detection of Antimicrobial Residues in Freshwater Aquaculture Fish in Erbil Governorate, Iraq

**DOI:** 10.3390/antibiotics13030225

**Published:** 2024-02-28

**Authors:** Dhary Alewy Almashhadany, Abdulwahed Ahmed Hassan, Rzgar Farooq Rashid, Amir Abdulmawjood, Izhar U. H. Khan

**Affiliations:** 1 Department of Medical Laboratory Science, College of Science, Knowledge University, Erbil 44001, Iraq; dhary.alewy@knu.edu.iq (D.A.A.); rzgar.faruq@knu.edu.iq (R.F.R.); 2 Metedi Medical Technology Distributions, Rathenaustraße 2, 35394 Giessen, Germany; a.hassan@metedi.de; 3 Department of Veterinary Public Health (DVPH), College of Veterinary Medicine, University of Mosul, Mosul 41002, Iraq; 4 Institute of Food Quality and Food Safety, University of Veterinary Medicine Hannover, Bünteweg 17, 30559 Hannover, Germany; amir.abdulmawjood@tiho-hannover.de; 5 Agriculture and Agri-Food Canada, Ottawa Research and Development Centre, 960 Carling Ave., Ottawa, ON K1A 0C6, Canada

**Keywords:** antimicrobial residues, freshwater aquaculture fish, Qualitative Field Disk Assay (QFDA), Disk Diffusion Assay (DDA)

## Abstract

The excessive and uncontrolled application of antibiotics in the fish farming industry, coupled with a lack of health monitoring and medication practices, is a driving force behind the escalating development of antimicrobial resistance. The present study assessed and compared qualitative field diffusion (QFD) and disk diffusion (DD) assays for the detection of antimicrobial residues (ARs) in diverse freshwater aquaculture fish. A total of 380 freshwater aquaculture fish (160 fresh and 180 frozen) samples were systematically collected between January and June 2021 from various retail stores located in Erbil Governorate, Iraq. Based on QFDA results, overall, ARs were detected (52; 15.3%) at a relatively lower frequency with comparatively higher frequency (21; 31.1%) in fresh than (31; 17.2%) frozen fish samples. On the other hand, DDA also revealed a comparable (45; 13.2%) prevalence rate of ARs. However, a low detection was observed more in fresh (17; 10.6%) than frozen (28; 15.6%) fish samples. Moreover, no statistically significant disparity (χ^2^ = 0.069; *p* = 0.79) between two assays and types of fish was recorded. In conclusion, the results of the present study showed that detecting a considerable frequency of ARs in these fish samples raises concerns about potential threats to public health. This underscores the necessity for understanding antibiotic application in aquaculture and its potential connection to antibiotic resistance in bacterial pathogens. Such comprehension is pivotal for formulating and implementing effective control and farm management strategies to address this pressing issue.

## 1. Introduction

Antimicrobial agents are used in livestock and aquaculture for therapeutic, meta- and prophylaxis purposes [1]. The intensive practices of aquaculture, adopted to meet the growing demand for fish, have shown evidence of antimicrobial agents mixed in feed for disease prevention [2]. Largely used antibiotics that fish do not effectively metabolize may excrete back into the environment where an estimated 75% of the antibiotic residues (ARs) fed to fish are excreted into the water [3,4]. Nonetheless, the persistence of ARs, under “One Health Continuum” in fish is a global health concern [5].

The incidence of ARs increases when fish are harvested for human consumption while still on medication or shortly after medication prior to the elapse of the withdrawal period (WP) [6]. The consumption of such fish may result in human health risks and conditions such as drug hypersensitivity reactions, the disruption of normal intestinal flora and carcinogenic, mutagenic and teratogenic effects [6,7,8]. Along with health concerns, the development and propagation of antimicrobial resistance may occur in fish-associated microorganisms and the horizontal gene transfer of resistance genes to other bacteria; whereas low-level doses of antibiotics in food products consumed for long periods may also lead to an increase in antimicrobial resistance in bacterial species and strains [7,8,9,10].

The presence of ARs in aquaculture, specifically fish and shrimp, has become a subject of concern in recent years [11,12,13,14]. Antibiotics are widely used in aquaculture to prevent and treat bacterial infections. Several studies have reported the presence of ARs in aquatic products where antibiotics such as quinolone and sulphonamide are the most detected antibiotics in fish and shrimp, with a higher concentration and detection frequency [9,15,16,17,18]. However, the excessive and unrestricted use of antibiotics can lead to the presence of ARs in seafood products, posing potential risks to human health and environment, potentially increase in the prevalence of some non-communicable diseases such as cancer. Moreover, it has been reported that an increase in antibiotic-resistant bacteria is associated with antibiotic use in aquaculture [17,18,19].

Various analytical techniques have been used to detect and quantify the presence of antibiotics. The most detected antibiotics in aquatic products include tetracyclines, fluoroquinolones, sulfonamides and chloramphenicol. Tetracycline residues are frequently found in fish and shrimps due to their broad-spectrum effectiveness against a wide range of bacterial pathogens [20]. Excessive use of tetracyclines can result in the accumulation of residues in aquatic products. Fluoroquinolones, used to treat bacterial infections in aquaculture, have been associated with the development of antibiotic resistance and have been reported at high levels in water, sediment and aquaculture organisms [12,21].

Sulfonamides, frequently used antimicrobial agents in aquaculture, have raised concerns due to their presence as residues in fish and shrimp, which pose potential health risks and contribute to the development of antibiotic resistance. Similarly, chloramphenicol, a broad-spectrum antibiotic banned in many countries due to its adverse effects on human health, has been detected in some fish and shrimp samples and can cause serious blood disorders in humans. Efforts are being made to regulate and control the use of antibiotics in aquaculture to minimize the presence of residues in aquaculture. Many countries have established maximum residue limits (MRLs) for antibiotics in fish and shrimps to ensure consumer safety. Regular monitoring and surveillance programs are implemented to enforce these regulations [12,22,23].

Methods for the surveillance testing of ARs have been classified into two categories: (a) qualitative screening tests; and (b) quantitative screening tests, where a quantitative method is more precise and considered a confirmatory assay. The qualitative screening test is based on microbial (e.g., *Micrococcus luteus* and *Bacillus subtilis*) growth inhibition; the inhibitory zones lacking bacterial colonies around the sample deposit sites indicate the potential presence of antibiotics. The test serves as a useful tool to prevent the entry of antibiotics into the food chain, but these tests are typically non-specific [24,25]. Microbiological or bioassay methods are usually the initial methods used to analyze a sample and establish the presence or absence of ARs [26,27,28]. In the European Union (EU), the most common surveillance programs for AR control are initiated with the high-throughput screening of samples using easy and inexpensive methods [29]. AR detection methods should be capable of detecting a broad spectrum of antimicrobials at regulatory levels, with ideally no more than 5% false compliant results. Furthermore, presumptive non-compliant results must be confirmed using a standard method [30]. Quantitative methods, such as high-performance liquid chromatography (HPLC), are used to identify and quantify specific ARs in samples that test positive during screening [31]. Both qualitative and quantitative methods must be sensitive to detect sublethal concentrations of antibiotics and require minimal technical expertise. Additionally, for field applications, factors such as cost, selectivity, biosafety and the ability to multiplex must be optimized and evaluated [32].

Based on these criteria, there is a lack of data, such as measurement of antibiotic concentrations in food, especially fish, with respect to the levels and concentrations of ARs in freshwater aquaculture fish in Iraq. Therefore, the aim of this study was to qualitatively estimate the ARs in fresh and frozen freshwater aquaculture fish and determine their rate of prevalence during the study period in Erbil Governorate, Iraq.

## 2. Results

### 2.1. Assessment of ARs by QFDA

Of the total 340 fish samples, 52 (15.3%) samples tested positive for the presence of ARs (Table 1). Statistically, the percentage of fish samples containing ARs ranged from 11.6 to 19.6% (95% CI). Table 1 presents the results of the inhibition zone measurements categorized into four categories: absence, weak, moderate and high. Among the positive samples, only 8 (5%) of the fresh and 12 (6.7%) of the frozen fish samples showed a high level of ARs. Most of the inhibition zones of both types of fish samples were categorized as weak and considered doubtful samples, and recorded as negative for ARs.

### 2.2. Assessment of ARs by DDA

As compared to QFDA data, only 45 (13.3%) samples showed as positive for the presence of ARs (Table 1). The percentage of fish samples containing ARs ranged from 9.8% to 17.3% (95% CI). Table 1 presents four levels (absence, weak, moderate and high) of inhibition zone measurement results. Of the total 45 positive samples, only 5 (3.1%) fresh and 11 (6.1%) frozen fish samples showed a high level of ARs. Most of the inhibition zones in both types of fish samples were categorized as weak, considered doubtful and recorded as negative results for ARs. However, statistically no significant (χ^2^ = 0.069; *p*-value = 0.79) difference between the QFDA and DDA methods in detecting ARs in fresh and frozen fish samples was observed.

### 2.3. Temporal Variations of ARs in Fish

**Figure 1:**
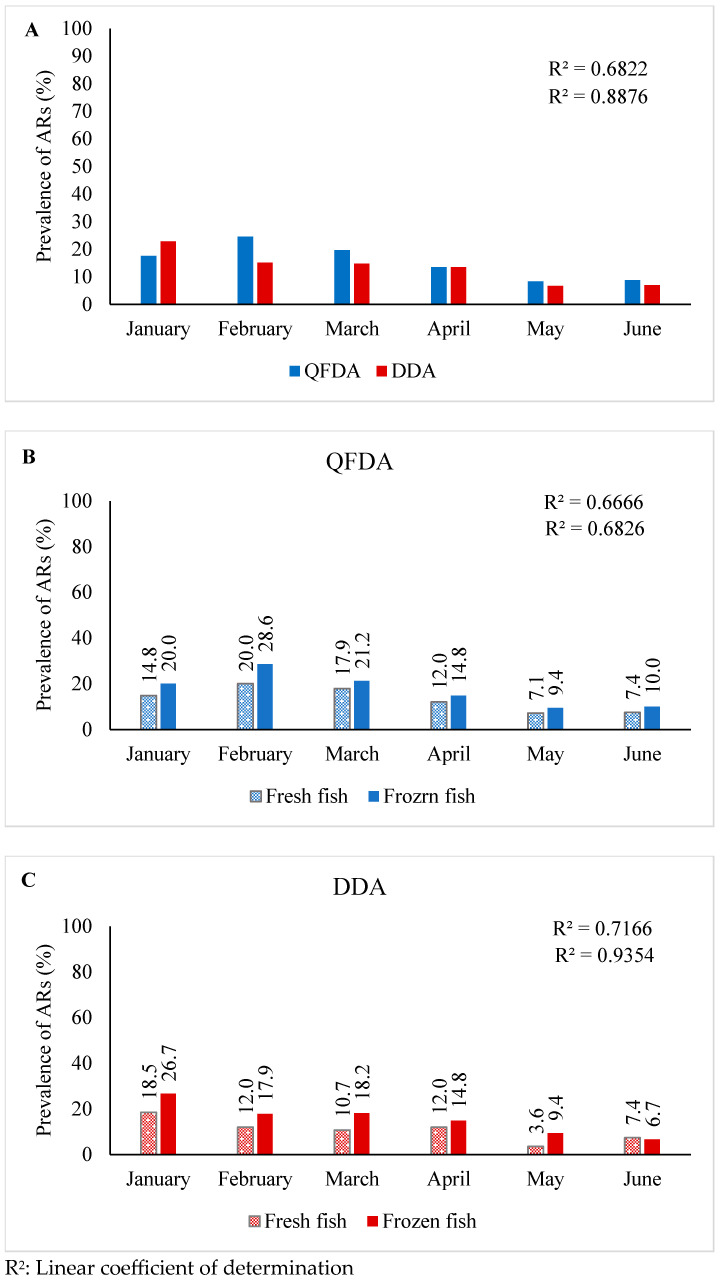
Panel (**A**): Monthly percentage of ARs prevalence in total fish samples assessed by QFDA and DDA, where positive percentage of samples is shown above each bar; Panel (**B**): Monthly percentage of positive ARs in fresh and frozen fish samples assessed by QFDA; Panel (**C**): Monthly percentage of positive ARs in fresh and frozen fish samples assessed by DDA.

Overall, ARs were detected in both types of fish and sources at variable frequencies during the sampling period. The QFDA showed the highest occurrence of ARs in February (24.5%), while the lowest was recorded in May (8.3%) (Table 2). Conversely, DDA results revealed the highest frequency of ARs in January (22.8%) as compared to May (6.7%) with the lowest rate of detection (Table 2). A robust association was observed, with a linear coefficient of determination (r^2^) of 0.865 for DDA as compared to 0.679 for the QFDA assay over the 6-month progression (Figure 1A).

The QFDA method detected higher ARs in frozen (r^2^ = 683) as compared to fresh (r^2^ = 667) (Figure 1B) fish samples. Similar results were recorded with DDA, where higher ARs (r^2^ = 935) were detected in frozen than in fresh (r^2^ = 717) fish samples (Figure 1C).

## 3. Materials and Methods

### 3.1. Fish Sampling

A total of 340 freshwater aquaculture fish, including 160 fresh (*Cyprinus carpio*, *Ctenopharyngodon Idella* and *Hypophthalmichthys molitrix*) and 180 frozen (primarily carp) samples, were collected between January and June 2021 from retail markets in Erbil governorate, Iraq. The samples were wrapped in sterile polythene bags, placed in a cooler with freezer packs, and transported to the Department of Medical Laboratory Science (DMLS). The fresh and frozen fish samples were stored at 4 °C and −20 °C, respectively, until further analysis.

### 3.2. Source and Type of Frozen Fish

The primary supply of frozen fish in the Iraqi fish market is imported from various countries, including Turkey, China, Vietnam, India, Thailand, Iran, the United Arab Emirates (UAE), Egypt and Saudi Arabia [33]. The predominant fish species imported from these countries are common carp (*C. carpio*), grass carp (*C. idella*) and silver carp (*H. molitrix*).

### 3.3. Inspection of Fresh Fish

All locally sourced fresh fish samples underwent a thorough inspection of various morphological features, including eye and skin color, where the absence of physical changes generally signifies the absence of disease as well as the color of the gills and the absence of mucus on the external body of the fish (absence of autoanalysis).

### 3.4. Detection of Antimicrobial Residues

#### 3.4.1. Qualitative Field Disk Assay (QFDA)

##### Preparation of Spore Suspension

The spore suspension of the *Bacillus subtilis* ATCC6633 reference strain, obtained from the Central Veterinary Laboratory (CVL) in Erbil, Iraq, was prepared for the assay as previously described by Almashhadany et al. [34]. Briefly, the cell density of bacterial suspension was adjusted to McFarland 0.5 standard, and a heavily grown inoculum of *B. subtilis* was subcultured on Nutrient agar plates (HiMedia, Thane, India) and incubated at 30 °C for 10 days to induce sporulation. For the elimination of vegetative cells, 15 colonies were harvested and suspended in 10 mL sterile 0.85% NaCl solution and heated at 70 °C for 10 min. Subsequently, the suspension was centrifuged at 1500× *g* for 3 min, and the pellet was washed with 10 mL sterile distilled water. The pellet was re-suspended in 1 mL of distilled water to obtain a pure endospore suspension. The final suspension was stored at 4 °C for future use.

##### Preparation of Test Plates

To prepare the test plates, the Muller-Hinton agar (HiMedia, India) was prepared following the manufacturer’s instructions. The agar medium was cooled in a water bath to 45 °C, maintaining its fluid consistency. Then, 0.1 mL of the final *B. subtilis* endospore suspension was added to 100 mL Muller-Hinton agar. The molten agar mixture was poured into Petri dishes and left to solidify at room temperature. The resulting Müller-Hinton Endospore agar (MHEA) plates were stored at 4 °C and used for the assay within one week. Moreover, for quality control, two separate sets of MHEA plates containing penicillin G sodium disks (10 IU) and sulfadimidine (0.5 μg) incubated at 37 °C for 24 h were also included.

##### Qualitative Field Disk Assay (QFDA)

The assay was conducted according to the protocol previously described [35,36]. Briefly, a sterile cork borer was used to prepare a disk-shaped sample with 2-mm thickness and 8-mm diameter dimensions. Subsequently, the disk-shaped sample was placed on the MHEA plate and aerobically incubated, without inversion, at 37 °C and the results were recorded after 24 h. A positive result was indicated by the presence of transparent inhibition zones around the disk, with a ≥1 mm diameter, indicating the presence of ARs in the fish sample. Conversely, samples showing <1 mm or no inhibition zone around the disk were considered negative for ARs. In addition, MHEA plates with a penicillin G sodium (10 IU) disk and without any added fish samples were used as positive and negative controls.

#### 3.4.2. Disk Diffusion Assay (DDA)

For DDA, the paper disks were initially prepared by cutting Whatman No.1 filter paper into approx. 1 cm in diameter and sterilized by autoclaving at 121 °C for 15 min. The DDA was performed by making five deep incisions in fish muscles, each measuring 2–3 cm in length, at different sites using a clean sterile surgical scalpel. Sterile paper disks were inserted into the incisions and allowed to saturate for 5 min. Five saturated disks were removed from muscle incisions using sterile forceps for each sample and placed on MHEA plates. The plates were then inverted and incubated at 37 °C for 24 h. After the incubation period, the transparent inhibition zones were measured, and the average was recorded according to previous classifications [37,38]. Samples with an average inhibition zone around the disk, measuring ≥2 mm in diameter, were considered positive for ARs, indicating the presence of antibiotics in the fish sample. Conversely, samples exhibiting a zone size ≤ 1 mm or an absence of an inhibition zone around the disk were categorized as negative. The concentration of ARs in the samples was classified into three categories based on the size of the inhibition zone: weak (>1 and <2 mm), moderate (>2 and <3 mm) and high (≥3 mm). The zone size around each positive sample was measured using a vernier caliper. Since no zones appeared in the control samples, the diameter of the zones in the samples was recorded for evaluation. Additionally, as positive and negative controls, MHEA plates with a penicillin G sodium (10 IU) disk and without any added fish samples were used.

### 3.5. Statistical Analysis

The two groups of fish samples were analyzed using McNemar Chi-square Contingency and Fisher’s Exact Tests to determine if there were significant differences between the fish sample types and the assays. The linear coefficient of determination (R^2^) was calculated between the period of the study, the type of samples, and the two AR detection assays. The significance level was set at *p* < 0.05. Additionally, the confidence interval (CI) test was employed to estimate the range of plausible values for study parameters, such as the sample mean and standard deviation. This estimation was based on a sample from the study, with a confidence level of 95% used for the CI.

## 4. Discussion

Previous research studies have established a clear link between the presence of ARs in fish and concerns about risks to public health and the environment [39,40]. In Iraq, there is a general lack of clarity regarding enforced policies and regulations concerning antibiotic usage in farm animals and freshwater fish aquaculture; however, there are a few studies that have directly and specifically investigated ARs in various food and food products, including fish, poultry, sheep and cow milk and raw beef in Iraq [41,42,43].

The unrestricted and excessive use of antibiotics by farmers, particularly fish breeders, along with the absence of health monitoring and proper medication practices, contributes to the escalation of bacterial resistance to antibiotics. In Iraq, there are problems in controlling the use of antibiotics, as well as the fact that farmers purchase and apply antibiotics without prescription. Therefore, in the present study, we investigated the ARs in fresh and frozen fish collected from retail markets in Erbil Governorate, Iraq. The results of the present study revealed a variable rate of AR prevalence in fresh and frozen fish using the QFDA and DDA methods. It is important to note that the results obtained from both assays are based on the size of the inhibition zone, which can be affected by various factors, including the type and concentration of antibiotics, the type and cell density of indicator bacteria and properties of the media, such as the pH and agar layer thickness [44]. In order to minimize the impact of media properties on the inhibition zone results of the samples, we performed quality control tests for each batch of MHEA plates. Therefore, samples showing weak inhibition zones observed in either assay may have been influenced by one or more of the above-mentioned factors and were classified as negative for ARs. The limitations of this study may include a lack of sensitivity to detect low levels of ARs, particularly in samples that showed weak inhibition zones. Additionally, the study may not distinguish between different types of antibiotics and concentrations since both methods are qualitative and may not provide precise quantitative values. When comparing the two methods, the results indicate that the QFDA method can be used for the screening of ARs in fish samples. However, caution is needed when comparing different qualitative methods used for the detection of ARs in fish and other food and food products.

Comparing our results with other studies, a similar rate of ARs frequency (11%), in Benin, with a significant (*p* < 0.05) difference between fish species, was reported [45]. However, a comparatively high frequency (25%) of ARs were detected in aquaculture fish and shrimp in Vietnam [11]. Similarly, in Turkey, a higher prevalence of ARs (33%) was observed in fish samples, primarily fluoroquinolones and tetracyclines [46]. Moreover, a surveillance study in Shanghai had a high (52.1%) rate of ARs in aquatic products, whereas, from 35 aquatic products, the overall detection frequency was 91.7%, ranging from 82% to 17% in snakeheads, loaches, carps, yellow-head catfishes and shrimps [47]. In comparison to our results, one study showed an overall high rate (24%) of AR prevalence in frozen, canned and processed fish, as well as frozen shellfish, whereas, among all sample types, a significantly high (88%) rate of AR was detected in canned fish samples [48]. Similarly, a high rate (45.6%) of ARs in various frozen fish types sourced from different countries was reported by Kukhtyn et al. [49]. Furthermore, a high frequency of nitrofurantoin (70%) and sulfite (43%) as compared to fluoroquinolone (17%) and oxytetracycline (7%) was observed in frozen shrimps [12]. However, in our study, a relatively higher frequency of ARs was recorded in the fresh than in the frozen fish, there was no statistical difference between the two methods and the fish types. This could possibly be due to the application of antimicrobials that lead to the higher rate of persistence of ARs in fresh fish than in frozen fish.

Although there is a lack of data on the seasonal prevalence and variation of ARs in fish, an acceptable conclusion can be drawn from previous studies where significant seasonal variations in the detection of ARs in fresh and marine water have been assessed [50,51]. Our study results, where the seasonal variation in ARs frequency was observed, indicate that the farmers may apply more antibiotics during winter compared to the summer months. Moreover, it is also possible that the antibiotics may degrade faster at a higher rate than at low temperatures. However, further research is warranted to understand the underlying environmental and chemical factors driving such variations and their potential implications for aquatic ecosystems and human health. The seasonality of bacterial infections in aquatic environments largely depends on climate and water salinity [52]. Moreover, pathogenic species of *Aeromonas*, *Shewanella* and *Vibrio* have been predominantly found in cultured fish during winter and spring [53,54]. Additionally, the fish industry may apply antibiotics for preservation purposes, especially when certain types of fish are only available during specific seasons in the year. Our study revealed that the highest occurrence of ARs was in February (24.5%), while the lowest was recorded in May (8.3%). Contrary to our results, the highest (27.74 µg/kg) and lowest (1.37 µg/kg) average antibiotic residual concentrations in Nile tilapia fish collected from the Rosetta branch of the Nile River were recorded in the spring and summer seasons. Interestingly, no ARs were detected during the autumn and winter seasons [50].

To control and mitigate the prevalence of ARs in animal source foods, including fish, several strategies have been developed, such as standard hygiene practices during fish rearing alongside the rational use of antimicrobials, which are considered effective approaches. Adherence to withdrawal periods should be accurately followed and enforced by relevant official authorities. Additionally, rapid, simple, sensitive and economical screening methods should be developed to examine ARs in food during production processes. Physical and chemical preservation techniques such as heat, refrigeration, UV irradiation, resins, charcoal and other preservatives are known to inactivate ARs. Whenever possible, replacements for antimicrobial drugs by biocontrol agents should also be considered [55,56].

## 5. Conclusions

Since the presence of ARs in food of animal origin is a public health concern, in this study, we reported on assessing and comparing QFDA and DDA methods for the detection and prevalence of ARs in fresh and frozen fish. Microbiological methods, particularly these assays, are quite suitable for the detection of ARs, especially as they are less expensive than immunochemical and chromatographic methods and can screen a large number of samples at minimal cost. No difference between the two methods was observed, which suggests that either method can be used in the analytical laboratory for the qualitative high-throughput screening of samples. The results suggest that the withdrawal period needs to be observed before fish are marketed. Moreover, the public needs to be aware of the appropriate cooking of fish for the degradation of ARs. In addition, the proper use of antimicrobial agents is required to prevent, treat and control diseases to ensure the effective implementation of regulatory measures. The results will help farmers and policymakers to develop or improve best management practices and regulations to reduce the uncontrolled use of antibiotics in aquaculture in Iraq. Moreover, it will also aid in reducing potential health risks and antibiotic resistance in humans and animals. Therefore, the implementation of these regulations would be useful in monitoring and controlling the production chain, promoting responsible antibiotic use practices and encouraging the development of alternatives to antibiotics for disease management and prevention.

## Figures and Tables

**Table 1:** Number (percent) and comprehensive correlation of ARs inhibition zone degrees in fresh and frozen fish samples using QFDA and DDA methods.

Methods	Fish	Number of Samples	ARs Inhibition Zone (mm) Degrees				Total Number of Positive (% **)	Statistical Values	
			Absence (0–≤1)	Weak * (>1–<2)	Moderate (>2–<3)	High (≥3)		95% CI	*p*-Value
QFDA	Fresh	160	128	11	13	8	21 (13.1)	8.3–19.4	0.295
	Frozen	180	122	27	19	12	31 (17.2)	12.0–23.6	
	Total	340	288	38	32	20	52 (15.3)	11.6–19.6	
DDA	Fresh	160	118	25	12	5	17 (10.6)	6.3–16.5	0.175
	Frozen	180	118	34	17	11	28 (15.6)	10.6–21.7	
	Total	340	236	59	29	16	45 (13.2)	9.8–17.3	

* Weak results were considered doubtful samples and recorded as negative for ARs. ** Moderate and high inhibition zones were considered positive for ARs.

**Table 2:** Monthly rate of prevalence including number and percentage of fish total samples and positive samples investigated for ARs using QFDA and DDA methods.

Method	Month	Fresh Fish		Frozen Fish		Total Samples of Both Fish Types	
		Number of Samples	Number of Positive (%)	Number of Samples	Number of Positive (%)	Total Number	Total Positive (%)
QFDA	January	27	4 (14.8)	30	6 (20)	57	10 (17.5)
	February	25	5 (20)	28	8 (28.6)	53	13 (24.5)
	March	28	5 (17.9)	33	7 (21.2)	61	12 (19.7)
	April	25	3 (12)	27	4 (14.8)	52	7 (13.5)
	May	28	2 (7.1)	32	3 (9.4)	60	5 (8.3)
	June	27	2 (7.4)	30	3 (10)	57	5 (8.8)
	Total	160	21 (13.1)	180	31 (17.2)	340	52 (15.3)
DDA	January	27	5 (18.5)	30	8 (26.7)	57	13 (22.8)
	February	25	3 (12)	28	5 (17.9)	53	8 (15.0)
	March	28	3 (10.7)	33	6 (18.2)	61	9 (14.7)
	April	25	3 (12)	27	4 (14.8)	52	7 (13.4)
	May	28	1 (3.6)	32	3 (9.4)	60	4 (6.6)
	June	27	2 (7.4)	30	2 (6.7)	57	4 (7.0)
	Total	160	17 (10.6)	180	28 (15.6)	340	45 (13.2)

## Data Availability

Data are contained within the article.
